# Adherence to the Healthy Low-Carbohydrate Diet and incident depression and anxiety

**DOI:** 10.3389/fpubh.2025.1746849

**Published:** 2026-01-16

**Authors:** Tian Qin, Yinfei Lu, Fenghuixue Liu, Zhongwei Zhang, Ping Yin

**Affiliations:** 1 Department of Epidemiology and Biostatistics, School of Public Health, Tongji Medical College, Huazhong University of Science and Technology, Wuhan, China; 2 Department of Geriatrics, WuHan Red Cross Hospital, Wuhan, China

**Keywords:** anxiety, depression, Healthy Low-Carbohydrate Diet, inflammation, UK Biobank

## Abstract

**Background:**

This study investigated the prospective associations between adherence to a Healthy Low-Carbohydrate Diet (HLCD) and incident depression, anxiety, and their comorbidity, and explored the mediating role of inflammation.

**Methods:**

This cohort study analyzed 173,207 participants from the UK Biobank. The HLCD score was calculated based on the intake of total carbohydrate, vegetable protein, and unsaturated fat. Cox proportional hazards models and restricted cubic splines were used to examine linear and non-linear associations, respectively. Mediation analyses assessed the contribution of inflammatory markers.

**Results:**

Compared with the lowest HLCD score group (scores 0–6), the lowest risks were observed in the third group (scores 13–18) for depression (hazard ratio [HR]: 0.705, 95% confidence interval [CI]: 0.626–0.793), the fifth group (scores 25–30) for anxiety (HR: 0.791, 95% CI: 0.688–0.909), and the second group (scores 7–12) for comorbidity (HR: 0.673, 95% CI: 0.555–0.816). Restricted cubic splines revealed non-linear, “L”-shaped associations between HLCD scores and the risks of all three outcomes (all P for non-linearity < 0.05). Mediation analyses indicated that white blood cell count, C-reactive protein, and the low-grade chronic inflammation score played statistically significant but weak mediating roles in these associations.

**Conclusions:**

Higher adherence to an HLCD is associated with reduced risks of depression, anxiety, and their comorbidity, potentially following a non-linear threshold pattern. While inflammation acts as a mediator, its modest contribution suggests that other biological mechanisms may also play pivotal roles in this protective relationship.

## Introduction

Mental disorders are increasingly recognized as one of the leading causes of the global health burden. In 2021, mental disorders accounted for 155 million disability-adjusted life-years (DALYs), ranking as the sixth leading cause of DALYs (1). Depression and anxiety, the most common mental disorders, ranked as the first and second leading causes of DALYs among mental disorders in 2021 (1). These two mental disorders are highly comorbid, and their comorbidity has a more significant impact than either condition alone (2, 3). Given the health impairment and disease burden caused by depression and anxiety, identifying risk factors and advancing primary prevention are urgent and worthwhile public health priorities.

Diet and nutrition, as key factors in both physical and mental health, should now be considered mainstream elements of psychiatric practice (4). Given that nutrients and foods are consumed in combination rather than in isolation, an analysis of dietary patterns is more appropriate, capturing the complexity of dietary intake (5). Healthy dietary habits, as an effective and low-risk intervention strategy, may have a significant impact on the prevention and treatment of depression and anxiety in individuals (6–8). Given the importance of macronutrient source and quality for human health, previous studies have established three low-carbohydrate diet (LCD) scores: overall (OLCD), healthy (HLCD), and unhealthy (ULCD) (9). Briefly, macronutrients were stratified into high- and low-quality carbohydrates, animal and plant proteins, and saturated and unsaturated fats (10). The “Healthy Low-Carbohydrate Diet score” was specifically derived from the intake of low-quality carbohydrate, vegetable protein, and unsaturated fat (11, 12). Specifically, the scoring system is bidirectional: protein and fat are scored positively (where higher intake yields a higher score), whereas carbohydrate intake is scored negatively/in reverse (where lower intake yields a higher score). Unlike the OLCD, the HLCD emphasizes the quality of these macronutrients, aiming to differentiate healthier patterns within low-carbohydrate diets. Several studies have evaluated the associations between low-carbohydrate diets (LCDs) and mental disorders, yielding inconsistent results. Systematic reviews indicated that LCDs showed no psychological benefit but might aid relapse prevention in treatment-resistant depression (TRD) (13, 14). Five studies conducted in specific populations suggested potential mental health benefits of LCDs (15–19). Another four cross-sectional studies, two from the United States, reported positive results, while two studies from Iran found no protective effects (20–23). However, these previous studies had limitations, including small sample sizes, a narrow focus on specific populations, an inability to infer causality due to cross-sectional designs, and a lack of mechanistic discussion.

The relationship between diet and mental health is likely mediated by complex biological pathways, with chronic low-grade inflammation playing a central role (24). Diets high in refined carbohydrates can trigger rapid fluctuations in blood glucose and insulin, promoting oxidative stress and systemic inflammation (25). Conversely, dietary patterns rich in unsaturated fats and vegetable proteins (key components of a healthy low-carbohydrate diet) possess potent anti-inflammatory properties (26). Furthermore, inflammation may act as a nexus linking the gut-brain axis and neuroplasticity to mental wellbeing. Unhealthy dietary habits can disrupt gut microbiota composition, compromising intestinal barrier integrity and allowing pro-inflammatory cytokines to enter the circulation (27). These inflammatory mediators can cross the blood-brain barrier, potentially altering neurotransmitter metabolism and downregulating Brain-Derived Neurotrophic Factor (BDNF), a key protein involved in neuroprotection and mood regulation (28). Thus, reducing inflammation via dietary optimization may be a pivotal mechanism for preventing depression and anxiety.

Hence, in this study, we aimed to investigate the longitudinal associations between adherence to the Healthy Low-Carbohydrate Diet and incident depression, anxiety, and their comorbidity in a large population-based cohort of UK adults, and to explore the potential mediating role of inflammation in these associations.

## Methods

### Study design and population

The UK Biobank is a large prospective cohort established to investigate the genetic and environmental determinants of health outcomes. Approved by the North West Multi-Centre Research Ethics Committee (REC reference: 11/NW/03820), the study recruited over 500,000 adults aged 37–73 years (99.5% aged 40–69 years) during 2006–2010 (29). All participants who provided written informed consent completed a touchscreen questionnaire and a face-to-face interview, and underwent physical measurements and biological sample collection at one of 22 assessment centers across Scotland, England, and Wales (30).

In the present study, we included participants who had completed the online 24-h diet recall questionnaire at least once. More than half of the cohort lacked 24-h dietary recall data and were excluded (*n* = 291,168). Participants were excluded if they met any of the following criteria: (1) withdrew from the study (*n* = 158); (2) had depression or anxiety at baseline (*n* = 25,488); (3) reported use of anxiolytics or antidepressants at baseline (*n* = 4,376). This rigorous criterion aimed to eliminate potential prevalent cases that might lack a formal diagnosis in medical records; (4) reported abnormal total energy intake (< 500 or > 3,500 kcal/day for female participants and < 800 or > 4,000 kcal/day for male participants) (*n* = 2,397) (31); or (5) had missing information on covariates (*n* = 5,349). After the above exclusions, there were 173,207 participants in our final analyses (Supplementary Figure 1). To investigate the mediating role of inflammation indicators in the associations between the HLCD score and depression, anxiety, and their comorbidity, participants who lacked immune biomarkers or had extreme outliers (defined as < quartile Q1 – 3 × interquartile range [IQR] or > Q3 + 3 × IQR) were further excluded (*n* = 22,233). The final immune subset had a sample size of 150,974.

### Healthy Low-Carbohydrate Diet score

Dietary intake was measured using the Oxford WebQ, a web-based 24-h dietary recall questionnaire (https://biobank.ndph.ox.ac.uk/showcase/refer.cgi?id=118240), which collects information on the types and quantities of foods consumed, including beverages and daily nutrient intake (32, 33). Initially, at the end of the UK Biobank recruitment process (2009–2010), 70,000 participants completed the Oxford WebQ as part of their baseline assessment center visits. In addition, participants were invited to complete four additional questionnaires via their home computers every three to four months across four different periods between February 2011 and June 2012. An average dietary intake was calculated to account for variations in dietary intake if participants completed dietary assessments more than once.

In the UK Biobank, the “Estimated food nutrients yesterday” category provides estimates of nutrient intake based on participants' responses to the diet questionnaire (34). In this category, it specifically includes the estimated intake of total carbohydrate (Field ID: 26013), vegetable protein (Field ID: 26006), monounsaturated fatty acids (Field ID: 26032), n-3 fatty acids (Field ID: 26015), n-6 fatty acids (Field ID: 26016), and energy (Field ID: 26002) from overall diet from past 24 hours. The intake of unsaturated fat is equal to the sum of monounsaturated fatty acids, n-3 fatty acids, and n-6 fatty acids. These nutrient quantities are directly available within the dataset, requiring no further calculation from specific food composition data (34). Due to data availability constraints within the UK Biobank, we constructed a modified index adapted from the original HLCD. Instead of differentiating carbohydrate quality, our scoring method utilized total carbohydrate intake. To reflect the 'healthy' aspect of the dietary pattern, we emphasized the substitution of carbohydrate with high-quality vegetable protein and unsaturated fat.

Participants were stratified into 11 specific strata according to the distribution of energy intake percentages from total carbohydrate, vegetable protein, and unsaturated fat. For the percentage of energy from vegetable protein and unsaturated fat, individuals in the highest stratum received a score of 10, and those in the lowest stratum received a score of 0. Conversely, for the percentage of energy from total carbohydrate, the scoring order was reversed. The scores for carbohydrate, vegetable protein, and unsaturated fat were then summed to compute the Healthy Low-Carbohydrate Diet (HLCD) score (ranging from 0 to 30) (9, 12). The specific ranges for each of the 11 strata are detailed in Supplementary Table 1. Accordingly, elevated scores reflect reduced carbohydrate intake and increased protein and fat intake. In our study, participants were divided into five groups for analysis based on equal intervals of the HLCD score.

### Outcome identification

Depression and anxiety outcomes were identified using data on ‘first occurrences' of health-related outcomes in the UK Biobank. This category represents the earliest recorded date of diagnosis, identified by aggregating relevant ICD codes across medical records (hospital, primary care, death registries, and self-reports (https://biobank.ndph.ox.ac.uk/showcase/refer.cgi?id=593). Follow-up occurrences of depression and anxiety were confirmed by the International Statistical Classification of Diseases and Related Health Problems, Tenth Revision (ICD-10) codes F32-F33 and F40-F48, respectively (35, 36). In this study, comorbidity was defined as the incident occurrence of both depression and anxiety in the same participant at any point during the follow-up period, regardless of the time interval between the two diagnoses. For the survival analysis, the “date of event” for comorbidity was defined as the date of diagnosis of the second condition (i.e., the later of the two dates). The follow-up period for participants was calculated from the date of the baseline assessment to the date of the first occurrence of any of the following: death, loss to follow-up, the occurrence of the outcomes, and the last follow-up (October 31, 2022, for England; August 31, 2022, for Scotland; May 31, 2022, for Wales).

### Inflammation index

To explore the role of inflammation in the associations between the HLCD score and depression, anxiety, and comorbidity, we selected immune indices that capture different dimensions of the inflammatory response. Based on established links between diet and mental health, we included C-reactive protein (CRP) as a marker of systemic inflammation, and leukocyte parameters (lymphocyte count, neutrophil count, platelet count, white blood cell count) to reflect cellular immune activation. Additionally, the neutrophil-to-lymphocyte ratio (NLR) was included as a composite marker. To ensure the analysis focused on chronic low-grade inflammation rather than acute inflammatory states, extreme values of the inflammation indicators (< Q1 – 3 × IQR or > Q3 + 3 × IQR) were considered indicative of acute infection, severe trauma, or measurement error and were therefore excluded (37).

To comprehensively assess the level of inflammation, we calculated the low-grade chronic inflammation (INFLA) score, which has been reported to be associated with diet and mental health (38, 39). The four components of the INFLA score (CRP, WBC, platelets, and NLR) are recognized pro-inflammatory markers (40). For each component, participants were stratified into deciles based on the distribution of the study population. A scoring system was then applied to these deciles: the lowest four deciles (1st to 4th) were assigned negative scores ranging from−4 to−1, respectively; the highest four deciles (7th to 10th) were assigned positive scores ranging from +1 to +4, respectively; and the middle deciles (5th and 6th) were assigned a score of 0. The scores for the four components were summed to yield a total INFLA score ranging from−16 to +16, with higher scores indicating elevated levels of low-grade chronic inflammation (40).

### Assessment of covariates

Covariates consisted of sociodemographic characteristics, lifestyle factors, and other potential confounding factors. Sociodemographic covariates included age, sex, ethnicity, and the Townsend deprivation index (41). Lifestyle covariates included smoking status, frequency of alcohol intake, physical activity, and body mass index (BMI). Other covariates include the history of diabetes and hypertension as well as total energy intake. Covariates (except total energy intake) were collected at baseline (2006-2010). Total energy intake was assessed by the 24-h dietary recall questionnaire. A full description of covariates can be found in Supplementary Methods 1.

### Statistical analysis

For baseline characteristics, continuous variables were summarized as mean ± SD, while categorical variables were expressed as frequencies (percentages). Cox proportional hazards models were used to investigate the associations between the HLCD score and incident depression, anxiety, and their comorbidity, using follow-up time as the timescale variable. The results were expressed as HRs and their 95% CIs. To explore non-linear associations, the HLCD score was fitted using restricted cubic splines with four nodes (42). The median value (15) of the HLCD score was selected as the reference point (HR = 1) for the restricted cubic spline analyses. This reference was chosen to ensure the statistical stability of the risk estimates, as the variance of the regression coefficient is typically smallest at the centroid of the data distribution.

To explore how the full range of the HLCD score (from 0 to 30) affected the outcomes, participants were divided into five groups for analysis based on the equal interval grouping of the HLCD score ( ≤ 6, 7–12, 13–18, 19–24, and ≥ 25). Compared with grouping by quantiles, equal-interval grouping can better reflect the changes in the degree of adhering to a low-carbohydrate diet. The lowest group served as the reference. In addition, the HLCD score was entered into the Cox models as a continuous variable (per 5-point increment) to complement the grouped analysis results and assess potential non-linear associations. Three Cox models were fitted: Model 1 was unadjusted; Model 2 was adjusted for age, sex, Townsend scores, and ethnicity; Model 3 was additionally adjusted for smoking status, alcohol intake, BMI, physical activity, hypertension, diabetes, and total energy intake on Model 2.

A three-step approach was employed to explore the longitudinal mediating effect of inflammation in the associations above. First, multiple linear regression models were used to explore the influence of the HLCD score on single inflammatory factors. Second, we used Cox models to explore the effects of single inflammatory factors on depression, anxiety, and their comorbidity. Finally, mediation analyses were conducted to assess the role of inflammatory indicators in the associations between the HLCD score and the outcomes. The mediation proportions (95% CIs) were calculated only if the total effect was significant. All inflammatory indicators, excluding the INFLA score, underwent a natural logarithmic transformation. Standardization was applied to all indicators before the analytical procedures.

We conducted five sensitivity analyses to evaluate the robustness of our main results. First, we excluded participants who completed the 24-h dietary recall questionnaire only once because the data might not reflect habitual intake. Second, since the four additional online dietary questionnaires were conducted after baseline, the main analysis was repeated by using the follow-up time calculated from the latest dietary questionnaire. Third, cases of depression, anxiety, and their comorbidity occurring within the first five years of follow-up were excluded to eliminate the potential effects of reverse causation. Fourth, we explored the different associations of three components of the HLCD score (percentages of energy from low-quality carbohydrate, vegetable protein, and unsaturated fat) with the outcomes. Fifth, stratified analyses were performed based on age, sex, ethnicity, smoking status, alcohol intake, BMI, physical activity, hypertension, and diabetes to examine the association across subgroups. By adding interaction terms between the HLCD score and the stratification variables above in the model, the multiplicative interaction effects were evaluated.

Statistical analyses were conducted using SAS version 9.4 and R version 4.4.2. All statistical tests were two-sided, with a *P*-value of < 0.05 defining statistical significance.

## Results

### Baseline characteristics

The baseline characteristics of participants are presented in Table 1. A total of 173,207 participants were included in the study, with a mean age (standard deviation, SD) of 56.14 (7.97) years at baseline, among them, 80,766 (46.63%) were male. During a median follow-up (interquartile range, IQR) of 13.27 (12.68–14.08) years, 5,284 (3.05%) incident depression cases, 7,243 (4.18%) anxiety cases, and 1,713 (0.99%) comorbid cases were identified. Participants in this study had a Healthy Low-Carbohydrate Diet (HLCD) score ranging from 0 to 30, with a median (IQR) of 15 (11–19). Participants with higher HLCD scores were more likely to be female, have lower Townsend deprivation indices, be non-smokers, engage in higher levels of physical activity, maintain a normal BMI, have higher total energy intake, have diabetes, be free of hypertension, and exhibit slightly lower levels of systemic inflammation indicators.

**Table 1:** Baseline characteristics of the study participants stratified by categories of the HLCD score (range, 0–30).

**Characteristics**	**Total**	**Categories of the HLCD score**				
		≤ **6**	**7–12**	**13–18**	**19–24**	≥**25**
**Eligible participants for analysis** ^a^						
Age (years)	56.14 ± 7.97	55.95 ± 8.25	56.47 ± 8.00	56.19 ± 7.97	55.87 ± 7.90	55.33 ± 7.85
Sex (male, %)	80,766 (46.63)	4,150 (46.27)	24,344 (48.77)	32,189 (47.46)	16,432 (43.08)	3,651 (43.70)
**Ethnic (%)**						
White	165,794 (95.72)	8,304 (92.59)	47,737 (95.63)	65,336 (96.34)	36,595 (95.93)	7,822 (93.63)
Others	7,413 (4.28)	665 (7.41)	2,179 (4.37)	2,485 (3.66)	1,552 (4.07)	532 (6.37)
Townsend score (%)	−1.64 ± 2.83	−1.49 ± 2.96	−1.73 ± 2.80	−1.69 ± 2.81	−1.55 ± 2.86	−1.31 ± 2.99
**Smoking status (%)**						
Never smoked	99,382 (57.38)	5,275 (58.81)	29,554 (59.21)	38,908 (57.37)	21,244 (55.69)	4,401 (52.68)
Former smoker	61,139 (35.30)	2,894 (32.27)	16,893 (33.84)	24,001 (35.39)	14,075 (36.90)	3,276 (39.21)
Current smoker	12,686 (7.32)	800 (8.92)	3,469 (6.95)	4,912 (7.24)	2,828 (7.41)	677 (8.10)
**Alcohol intake (%)**						
Drink infrequently	88,011 (50.81)	5,702 (63.57)	26,679 (53.45)	32,715 (48.24)	18,699 (49.02)	4,216 (50.47)
Drink frequently	85,196 (49.19)	3,267 (36.43)	23,237 (46.55)	35,106 (51.76)	19,448 (50.98)	4,138 (49.53)
**Physical activity (%)**						
Low	36,576 (21.12)	1,960 (21.85)	10,454 (20.94)	14,589 (21.51)	7,999 (20.97)	1,574 (18.84)
Moderate	90,060 (52.00)	4,440 (49.50)	25,793 (51.67)	35,275 (52.01)	20,154 (52.83)	4,398 (52.65)
High	46,571 (26.89)	2,569 (28.64)	13,669 (27.38)	17,957 (26.48)	9,994 (26.20)	2,382 (28.51)
**Body mass index (kg/m** ^2^ **)**						
Underweight	924 (0.53)	40 (0.45)	237 (0.47)	361 (0.53)	237 (0.62)	49 (0.59)
Normal weight	65,253 (37.67)	3,096 (34.52)	18,372 (36.81)	25,556 (37.68)	14,808 (38.82)	3,421 (40.95)
Overweight	72,420 (41.81)	3,957 (44.12)	21,474 (43.02)	28,348 (41.80)	15,252 (39.98)	3,389 (40.57)
Obesity	34,610 (19.98)	1,876 (20.92)	9,833 (19.70)	13,556 (19.99)	7,850 (20.58)	1,495 (17.90)
Hypertension (%)	87,948 (50.78)	4,552 (50.75)	25,936 (51.96)	34,621 (51.05)	18,930 (49.62)	3,909 (46.79)
Diabetes (%)	7,220 (4.17)	260 (2.90)	1,732 (3.47)	2,785 (4.11)	1,917 (5.03)	526 (6.30)
Total energy intake (kcal/day)	2048.13 ± 542.37	1,914.96 ± 549.04	1,985.17 ± 533.88	2,081.60 ± 533.10	2,090.33 ± 544.27	2,102.82 ± 591.28
**Immune subset** ^b^ **, inflammation** ^c^						
Lymphocyte count	0.61 ± 0.30	0.63 ± 0.30	0.61 ± 0.30	0.60 ± 0.30	0.61 ± 0.29	0.60 ± 0.30
Neutrophil count	1.35 ± 0.32	1.36 ± 0.33	1.35 ± 0.32	1.35 ± 0.32	1.35 ± 0.32	1.34 ± 0.31
Platelet count	5.48 ± 0.23	5.49 ± 0.23	5.48 ± 0.24	5.48 ± 0.23	5.49 ± 0.23	5.48 ± 0.24
White blood cell count	1.86 ± 0.24	1.88 ± 0.24	1.87 ± 0.24	1.86 ± 0.24	1.86 ± 0.24	1.85 ± 0.24
CRP	0.09 ± 0.92	0.18 ± 0.92	0.11 ± 0.92	0.09 ± 0.92	0.07 ± 0.93	−0.01 ± 0.94
NLR	0.74 ± 0.38	0.73 ± 0.39	0.74 ± 0.38	0.75 ± 0.38	0.74 ± 0.38	0.74 ± 0.37
INFLA score	−0.88 ± 5.85	−0.53 ± 5.88	−0.83 ± 5.85	−0.88 ± 5.83	−0.94 ± 5.87	−1.30 ± 5.89

HLCD Healthy Low-Carbohydrate Diet, CRP C-reactive protein, NLR Neutrophil-to-Lymphocyte Ratio, INFLALow-grade chronic inflammation ^a^Eligible participants for analysis: (N = 173,207) ^b^Immune subset: (N = 150,974) ^c^Inflammation were natural log transformed except INFLA score.

Associations of the Healthy Low-Carbohydrate Diet score (range, 0–30) with incident depression and anxiety.

The results of the Cox proportional hazards models are presented in Table 2. Compared with the lowest reference group of the HLCD score (0–6), participants in the second (7-12), third (13-18), fourth (19-24), and fifth (25-30) groups had HRs (95 % CIs) of 0.785 (0.701–0.879), 0.706 (0.632–0.79), 0.705 (0.626–0.793), and 0.734 (0.626–0.86) for depression; 0.833 (0.753–0.922), 0.808 (0.732–0.892), 0.794 (0.715–0.881), and 0.791 (0.688–0.909) for anxiety and 0.756 (0.622–0.919), 0.673 (0.555–0.816), 0.687 (0.561–0.842), and 0.701 (0.532–0.925) for comorbidity. For every 5-point increment in the HLCD score, the HRs (95% CIs) were 0.936 (0.913–0.960), 0.958 (0.938–0.979), and 0.932 (0.893–0.974) for depression, anxiety, and comorbidity, respectively.

**Table 2:** Associations between the Healthy Low-Carbohydrate Diet score (range, 0-30) and risks of depression and anxiety.

**Category**	**N_case_/N_total_**	**Model 1** ^**a**^		**Model 2** ^**b**^		**Model 3** ^**c**^	
		**HR (95% CI)**	***P*** **-value**	**HR (95% CI)**	***P*** **-value**	**HR (95% CI)**	***P*** **-value**
**Depression**							
**Categories of the HLCD score**							
≤ 6	372/8,969	REF		REF		REF	
7–12	1,567/49,916	0.747 (0.667–0.836)	<0.001	0.760 (0.679–0.852)	<0.001	0.785 (0.701–0.879)	<0.001
13–18	1,949/67,821	0.681 (0.609–0.761)	<0.001	0.686 (0.614-0.766)	<0.001	0.706 (0.632-0.790)	<0.001
19–24	1,135/38,147	0.705 (0.627-0.792)	<0.001	0.692(0.616-0.779)	<0.001	0.705 (0.626–0.793)	<0.001
≥ 25	261/8,354	0.743 (0.634–0.870)	< 0.001	0.722(0.616-0.846)	<0.001	0.734 (0.626–0.860)	<0.001
5-point increment in the HLCD score	5,284/173,207	0.948 (0.924–0.971)	<0.001	0.937 (0.914–0.961)	<0.001	0.936 (0.913–0.960)	<0.001
**Anxiety**							
**Categories of the HLCD score**							
≤ 6	463/8,969	REF		REF		REF	
7–12	2,079/49,916	0.797 (0.720–0.881)	<0.001	0.812(0.734-0.898)	<0.001	0.833 (0.753-0.922)	<0.001
13–18	2,771/67,821	0.778 (0.705–0.859)	<0.001	0.786 (0.712–0.867)	<0.001	0.808 (0.732-0.892)	<0.001
19–24	1,583/38,147	0.790 (0.713–0.877)	<0.001	0.777 (0.700-0.861)	<0.001	0.794 (0.715-0.881)	<0.001
≥ 25	347/8,354	0.793 (0.690–0.912)	0.001	0.776 (0.675-0.892)	<0.001	0.791 (0.688-0.909)	<0.001
5-point increment in the HLCD score	7,243/173,207	0.967 (0.947–0.988)	0.002	0.957 (0.937–0.977)	<0.001	0.958 (0.938-0.979)	<0.001
**Comorbidity**							
**Categories of the HLCD score**							
≤ 6	127/8,969	REF		REF		REF	
7–12	508/49,916	0.710 (0.584–0.862)	<0.001	0.725 (0.597–0.881)	0.001	0.756 (0.622–0.919)	0.005
13–18	622/67,821	0.637 (0.526–0.771)	<0.001	0.643 (0.531–0.778)	<0.001	0.673 (0.555-0.816)	< 0.001
19–24	372/38,147	0.677 (0.553–0.828)	<0.001	0.663 (0.542–0.811)	<0.001	0.687 (0.561-0.842)	< 0.001
≥ 25	84/8,354	0.700 (0.531–0.922)	0.011	0.678 (0.515–0.894)	0.006	0.701 (0.532–0.925)	0.012
5-point increment in the HLCD score	1,713/173,207	0.941 (0.901–0.983)	0.006	0.929 (0.890–0.970)	<0.001	0.932 (0.893–0.974)	0.002

HLCD Healthy Low-Carbohydrate Diet. ^a^Model 1: unadjusted. ^b^Model 2: adjusted for age, sex, Townsend scores and ethnicity. ^c^Model 3: additionally adjusted for smoking status, alcohol intake, BMI, physical activity, hypertension, diabetes, and total energy intake on Model 2.

**Figure 1:**
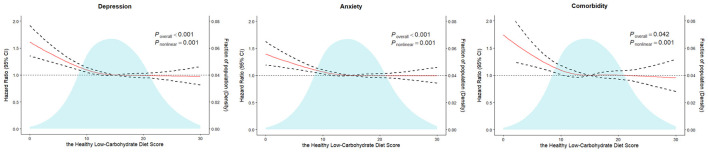
Restricted cubic spline regression models of the associations between the HLCD score and risks of depression, anxiety, and comorbidity. The model was adjusted for the same confounding variables as Model 3 presented above. HR, hazard ratio; CI, confidence interval; HLCD, Healthy Low-Carbohydrate Diet.

To further explore the shape of the dose-response relationship for the aforementioned associations, restricted cubic spline analyses were performed. The RCS analysis showed an ‘L'-shaped association for all three outcomes (Figure 1; all P for non-linearity < 0.05). The risks for all three outcomes decreased sharply with increasing HLCD score until approximately 15 points; beyond a score of 15, the declining trend in risk plateaued.

### Mediation analyses of inflammation

**Figure 2:**
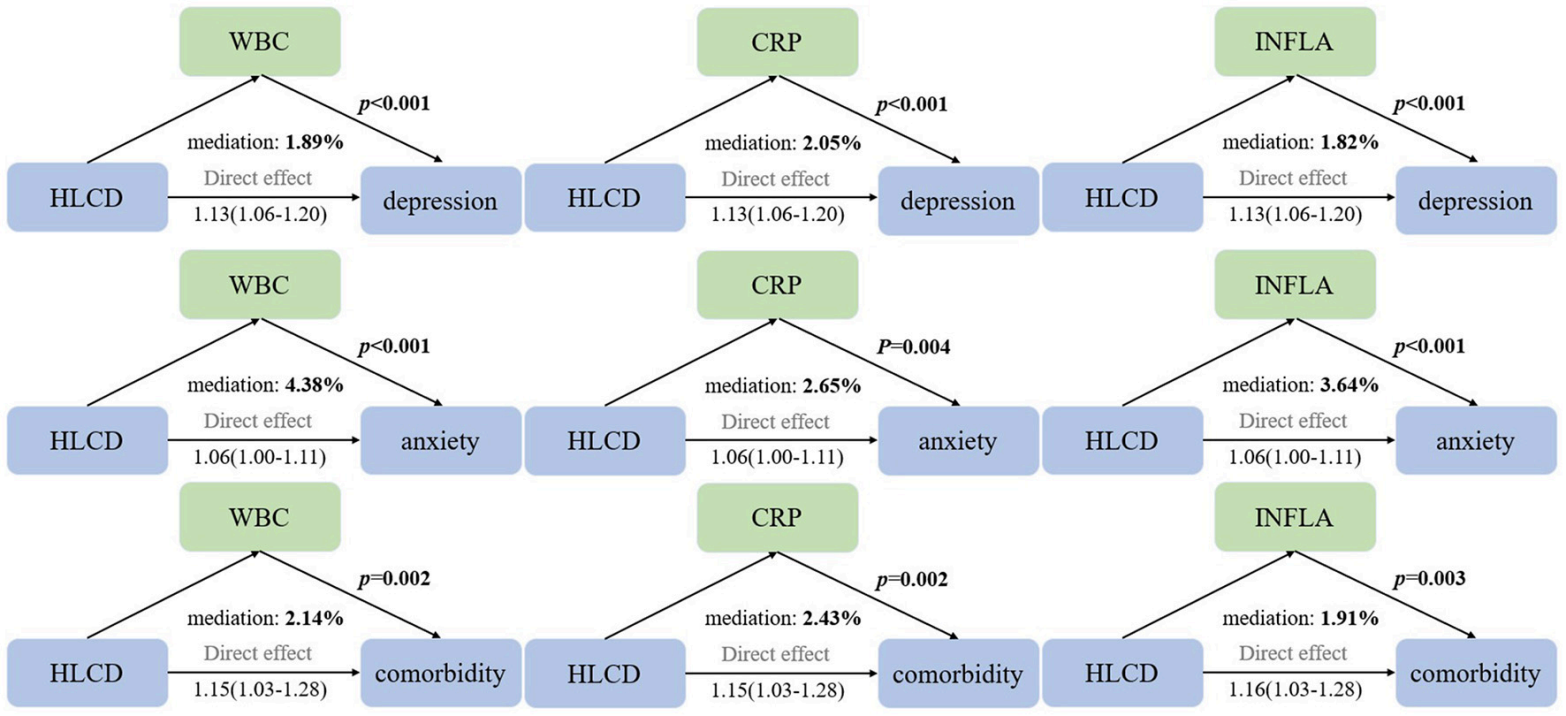
Mediation analyses with inflammatory indicators between the association of the HLCD score and risks of depression and anxiety among the immune subset (*N* = 150,974). Adjusted for age, sex, Townsend scores, ethnicity, smoking status, alcohol intake, BMI, physical activity, hypertension, diabetes, and total energy intake. HLCD, Healthy Low-Carbohydrate Diet; WBC, white blood cell; CRP, C-reactive protein; INFLA, Low-grade chronic inflammation score.

After full adjustment in Model 3, the HLCD score showed negative correlations with most inflammatory indicators except the neutrophil-to-lymphocyte ratio (NLR). Although associations with platelet and lymphocyte counts were non-significant, other indicators demonstrated significant associations with increased risks of depression, anxiety, and comorbidity (Supplementary Tables 8, 9). The mediation analyses indicated that the WBC count, CRP, and the INFLA score played significant mediating roles in the associations between the HLCD score and all three outcomes. The direct and indirect effects were consistent in direction, but the indirect effects were relatively small, suggesting weak mediation (Figure 2).

### Sensitivity and subgroup analyses

Within further analyses, we observed consistent inverse associations between the HLCD score and risks of incident depression, anxiety, and their comorbidity, including when excluding participants with only one valid 24-h dietary recall, redefining follow-up duration using the latest dietary assessment as baseline, and excluding cases occurring within the first 5 years of follow-up (Supplementary Tables 2–4). Supplementary Tables 5–7 display the associations of the three individual components of the HLCD score (percentages of energy from low-quality carbohydrate, vegetable protein, and unsaturated fat) with risks of incident depression, anxiety, and comorbidity. After full adjustment for covariates, for carbohydrate, only the highest intake quintile showed increased risks of depression and anxiety compared with the lowest reference quintile. For protein and fat, higher intake levels were generally associated with lower risks of all three outcomes, though the results for several quintile groups were not statistically significant. The inverse associations between the HLCD score and risks of depression and anxiety existed in most subgroups, but the results of the subgroup analysis were not statistically significant for comorbidity (Supplementary Figures 2–4). We also explored the potential interactions between the HLCD score and the subgroups above. We found that the protective effect of the HLCD score on depression was not significant in overweight participants; the protective effect on anxiety was not significant in normal-weight participants but was stronger in obese participants (Supplementary Figures 2–4).

## Discussion

In this large prospective cohort of UK adults, we found that greater adherence to the Healthy Low-Carbohydrate Diet (HLCD) was significantly associated with reduced risks of incident depression, anxiety, and their comorbidity, with all associations demonstrating non-linear dose-response relationships.

In recent years, a growing number of studies have investigated how a healthy diet affects depression and anxiety. Summarizing clinical trials and epidemiological evidence, a 2020 narrative review demonstrated that healthy dietary patterns that align with food and nutrition recommendations may help prevent and treat depression and anxiety disorders (43). More specifically, healthy dietary patterns that may confer psychological protection include, but are not limited to, the Mediterranean diet (44, 45), plant-based diet (46, 47), and the DASH diet (48, 49). Our findings confirm that adherence to the Healthy Low-Carbohydrate Diet (HLCD) was significantly associated with lower risks of depression, anxiety, and their comorbidity.

The Low-Carbohydrate Diet (LCD), first recommended as a nutritional intervention for epilepsy and later as a strategy for weight loss, now demonstrates possible efficacy in diabetes, heart disease, cancer, and lung/gut disorders, according to increasing evidence (50). Thus, our study extends prior evidence by demonstrating that greater adherence to the HLCD correlates with reduced risks of depression, anxiety, and their comorbidity, indicating the dual benefits of LCDs for both physical and mental health.

Previous research has highlighted the importance of the quality and source of macronutrients when adhering to LCDs (23). Compared with overall LCDs (OLCDs) and unhealthy LCDs (ULCDs), adherence to HLCDs may be more effective in improving depressive symptoms (23). To explore the dietary pattern that benefits people more, we selected the HLCD score and its three macronutrient components (percentages of energy from low-quality carbohydrate, vegetable protein, and unsaturated fat) were selected as our exposure factors.

Based on the dose-response relationships, we found that increasing the HLCD score from low to moderate significantly decreased the risks of depression, anxiety, and their comorbidity. However, increasing the HLCD score from moderate to high provided no additional benefits. The results of the main analysis also supported this nonlinear trend; specifically, using the lowest HLCD score group as the reference, participants with moderate HLCD scores demonstrated significantly greater mental health improvements compared with those with high scores. The observed non-linear association, favoring moderate adherence, may be attributed to distinct physiological and behavioral mechanisms. Overconsumption of carbohydrates may promote a state of chronic systemic inflammation, a process primarily mediated by postprandial hyperglycemia (51). However, carbohydrate consumption also plays a role in mood regulation by modulating insulin levels to enhance the uptake of tryptophan into the brain, which serves as a precursor for serotonin production (52, 53). Consequently, extreme restriction of carbohydrate intake may disrupt this physiological pathway, potentially increasing the risk of depression and anxiety by impairing serotonin synthesis. Behaviorally, moderate adherence represents a flexible and sustainable approach, while extreme adherence may reflect rigid dietary restraint, which is strongly associated with psychological distress and increased cortisol levels (54, 55). Furthermore, strict dietary rules can lead to social isolation due to the inability to participate in normal social dining, creating a psychological burden that may offset the physiological benefits of a healthy diet (56). Thus, the findings suggest that moderate dietary modifications may yield more benefits for mental health than extreme dietary interventions when adopting a low-carbohydrate diet.

Subgroup analyses revealed notable heterogeneity across BMI categories. Specifically, the protective effect of the HLCD on depression was not statistically significant in overweight participants. Regarding anxiety, the association was strongest in obese participants, whereas no significant protection was observed in the normal-weight group. Obesity is increasingly recognized as a state of chronic low-grade inflammation, creating a specific ‘immuno-metabolic' subtype of depression that may be particularly responsive to dietary interventions targeting metabolic health (57). Reducing carbohydrate intake mitigates postprandial hyperglycemia and hyperinsulinemia, thereby dampening the inflammatory signaling pathways often upregulated in obesity (57). The null findings in the overweight group may reflect the biological heterogeneity of depression. Unlike obesity, where metabolic dysregulation is a dominant driver, depression in overweight individuals may be less dependent on inflammatory pathways (58). BMI is an imperfect proxy for metabolic health. The overweight category likely encompasses a substantial proportion of metabolically healthy individuals who may not exhibit the systemic inflammation targeted by the HLCD (59).

In the sensitivity analyses, compared with the reference group (lowest quintile of carbohydrate intake), only the highest quintile exhibited an increased risk, and for protein and fat, higher intake levels were generally associated with lower risks. The association results of the macronutrients are consistent with the previous cross-sectional study (22). Regarding the Healthy Low-Carbohydrate Diet (HLCD) score, since moderate carbohydrate intake is generally considered optimal for health, the progression from moderate to high HLCD scores inherently necessitates increasingly strict carbohydrate restriction. Consequently, the potential downsides of extreme carbohydrate deprivation might offset the benefits of improved macronutrient quality. This trade-off plausibly explains the non-linear pattern observed in our restricted cubic spline (RCS) analysis, where increasing the HLCD score from moderate to high levels provided no additional protective benefits. Conversely, regarding the specific risk of excessive carbohydrate intake, the observed ‘threshold effect' likely reflects a displacement phenomenon, where excessive consumption of carbohydrates significantly crowds out protective nutrients, such as vegetable proteins and unsaturated fats. This aligns with our main findings, further emphasizing that dietary quality and the balance of macronutrient substitution are more critical determinants of mental health than carbohydrate quantity alone.

In this study, the mediation analyses indicated that inflammation partially mediated the associations between the HLCD score and the three outcomes. Several studies have investigated the inflammatory processes linking LCDs and mental health. The LCD or Ketogenic Diet (KD) may have antidepressant and mood-stabilizing effects by improving antioxidant activity and reducing inflammation (60). A review also summarized the inflammatory pathways linking KD to mental disorders (61). Finally, future longitudinal and experimental studies are needed to validate these inflammatory pathways and explore other potential mediators, such as alterations in synaptic plasticity and the gut-brain axis, to fully elucidate how HLCD confers neuroprotection.

Our study addresses critical gaps in the existing literature through its robust prospective design and extensive sample size, which provide high statistical reliability. Crucially, we extended the analysis beyond phenotypic associations to investigate underlying biological pathways, offering novel empirical evidence for inflammation as a key mediator linking the HLCD to psychological wellbeing. Furthermore, the five instances of dietary assessments over a year reflected seasonal differences in dietary intake components, enabling us to calculate the average dietary intake as a proxy for habitual intake. Our study also has several potential limitations. First, a limitation of our study is the inability to differentiate carbohydrate quality due to the nature of the UK Biobank dataset. Consequently, we developed a new scoring index based on data availability, which incorporates total carbohydrate intake. However, the essence of the healthy low-carbohydrate diet is effectively captured by the quality of the replacement nutrients. By focusing on the substitution of carbohydrates with high-quality vegetable proteins and unsaturated fats, we believe this modified approach offers a valid and reliable proxy for examining the diet-mental health relationship. Second, although depression and anxiety cases were identified from multiple sources, the possibility of missed or delayed diagnoses cannot be entirely ruled out. Third, a substantial number of participants were excluded due to incomplete 24-h dietary recall data, introducing potential selection bias. While this may result in a “healthier” analytical sample compared to the general population, empirical evidence from the UK Biobank suggests that reliable exposure-outcome associations can still be established despite such selection pressures. Furthermore, we adjusted for a comprehensive set of confounders to mitigate the influence of sociodemographic disparities. Finally, measurement errors and recall bias in dietary assessments were unlikely to be completely eliminated, although the use of repeated 24-h dietary recall assessments helped to capture habitual intake.

## Conclusions

This prospective study showed that high adherence to the HLCD reduced the risks of depression, anxiety, and their comorbidity, highlighting that dietary optimization may lead to better psychological health. Given the weak mediation effects of inflammatory indicators, further research exploring the mechanisms underlying these associations is warranted.

## Data Availability

The data analyzed in this study is subject to the following licenses/restrictions: UK Biobank data are available to all researchers for health-related research and public interest through registration on the UK Biobank (www.ukbiobank.ac.uk). This research has been conducted using the UK Biobank Resource under Application Number 162275. Requests to access these datasets should be directed to Tian Qin, qtsunnysky123@163.com.
