# Supplementary material for: Adherence to the Healthy Low-Carbohydrate Diet and incident depression and anxiety
Source: Front Public Health. 2026 Jan 16;13:1746849. doi: 10.3389/fpubh.2025.1746849 (PMC12855048; doi:10.3389/fpubh.2025.1746849)
Supplement: Supplementary file 1 [file Supplementary_file_1.docx]

**Supplementary material 1**

**Adherence to the Healthy Low-Carbohydrate Diet and incident depression and anxiety**

Tian Qin^a,1^, Yinfei Lu^b,1^, Fenghuixue Liu^a^, Zhongwei Zhang^a^, Ping Yin^a,*^

^a^Department of Epidemiology and Biostatistics, School of Public Health, Tongji Medical College, Huazhong University of Science and Technology, Wuhan, China

^b^Department of Geriatrics, WuHan Red Cross Hospital, Wuhan, China

*Correspondence to: Ping Yin, Department of Epidemiology and Biostatistics, School of Public Health, Tongji Medical College, Huazhong University of Science and Technology, 13 Hangkong Rd, Wuhan 430030 Hubei, China.

E-mail addresses: qtsunnysky123@163.com (Tian Qin), stelllnoob@outlook.com (Yinfei Lu), liufenghx@qq.com (Fenghuixue Liu), zhongweizzzz@163.com (Zhongwei Zhang), pingyin2000@126.com (Ping Yin).

^1^Tian Qin, Yinfei Lu contributed equally to this work as co-first authors.

**Supplementary Methods 1.** Assessment of covariates

**Supplementary Figure 1.** The flowchart of participant selection

**Supplementary Figure 2.** Subgroup analyses of associations between the Healthy Low-Carbohydrate Diet score and risk of depression.

**Supplementary Figure 3.** Subgroup analyses of associations between the Healthy Low-Carbohydrate Diet score and risk of anxiety.

**Supplementary Figure 4.** Subgroup analyses of associations between the Healthy Low-Carbohydrate Diet score and risk of comorbidity.

**Supplementary Table 1.** Criteria for determining the low-carbohydrate-diet (HLCD) score.

**Supplementary Table 2.** Associations between the HLCD score and risks of depression and anxiety after excluding participants who only completed the online 24-hour dietary recall questionnaire on 1 occasion (N=106,171).

**Supplementary Table 3.** Associations between the HLCD score and risks of depression and anxiety, redefining follow-up duration using the latest dietary assessment as baseline (N=171,610).

**Supplementary Table 4.** Associations between the HLCD score and risks of depression and anxiety after excluding depression or anxiety cases that occurred within the first five years of follow-up (N=169,484).

**Supplementary Table 5.** Associations between percentages of energy from low-quality carbohydrate and risks of depression and anxiety.

**Supplementary Table 6.** Associations between percentages of energy from vegetable protein and risks of depression and anxiety.

**Supplementary Table 7.** Associations between percentages of energy from unsaturated fat and risks of depression and anxiety.

**Supplementary Table 8.** Associations between the HLCD score and inflammatory indicators.

**Supplementary Table 9.** Associations between inflammatory indicators and risks of depression and anxiety.

**Supplementary Methods 1.**

**Assessment of covariates**

Covariates (except total energy intake) were collected at baseline (2006-2010). Covariates were made up of sociodemographic characteristics, lifestyle factors, and other potential confounding factors. Sociodemographic covariates included age (continuous), sex (males and females), ethnicity (classified as White and others), and the Townsend deprivation index [1]. The Townsend deprivation index, derived from the postcode of residence, was used to describe the area-based socioeconomic status through the quartiles of indices [2]. Lifestyle covariates, including smoking status (classified as never, former, and current smoking) and frequency of alcohol intake (divided into infrequent and frequent), were measured by self-report. Physical activity, the volume of which was calculated as the sum of walking, moderate, and vigorous activity over the previous week, measured as metabolic equivalents task (METs min/week) [3], was assessed by the International Physical Activity Questionnaire (IPAQ) short form. Physical activity was categorized into three groups: low (MET<600 min/week), moderate (600-3000 MET), and high (≥3000 MET). Hypertension was determined by the combination of antihypertensive medication use, records of diagnosis by doctors, and clinical measurement of blood pressure (systolic blood pressure (SBP)≥140 mmHg or diastolic blood pressure (DBP)≥90 mmHg). Diabetes was determined by the combination of medication for diabetes and records of diagnosis by doctors. Height and body weight were measured by well-trained nurses during the initial assessment center visit. Body mass index (BMI, kg/m2) was calculated as weight (kg) divided by height squared (m2) and divided into four categories according to the recommendations of the World Health Organization (WHO): underweight (<18.5 kg/m^2^), normal weight (18.5 to 25 kg/m^2^), overweight (25 to 30 kg/m^2^), and obesity (≥30 kg/m^2^). Total energy intake was assessed by the 24-hour dietary recall questionnaire. If participants completed dietary assessments multiple times, an average total energy intake was calculated. The details of these assessments can be found on the UK Biobank website (www.ukbiobank.ac.uk).

Eligible participants for analysis

N=173207

Exclude those with abnormal total energy intake (N=2397) or incomplete information of covariates

(N=5349)

N=210817

N=501985

N=180953

Exclude those with depression or anxiety (N=25488) or reported use of anxiolytics or antidepressants

(N=4376) at baseline

Exclude those who did not complete the 24h dietary recall questionnaire

at any occasion

N=291168

Exclude those who withdraw

from the survey

N=158

All participants

N=502143

Immune subset N=150974

Exclude those without immune biomarkers and extreme outliers (<Q1-3*IQR or >Q3+3*IQR)

**Supplementary Figure 1.** The flowchart of participant selection


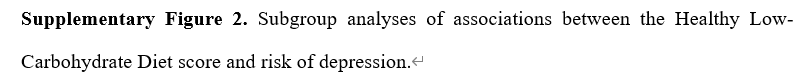
**
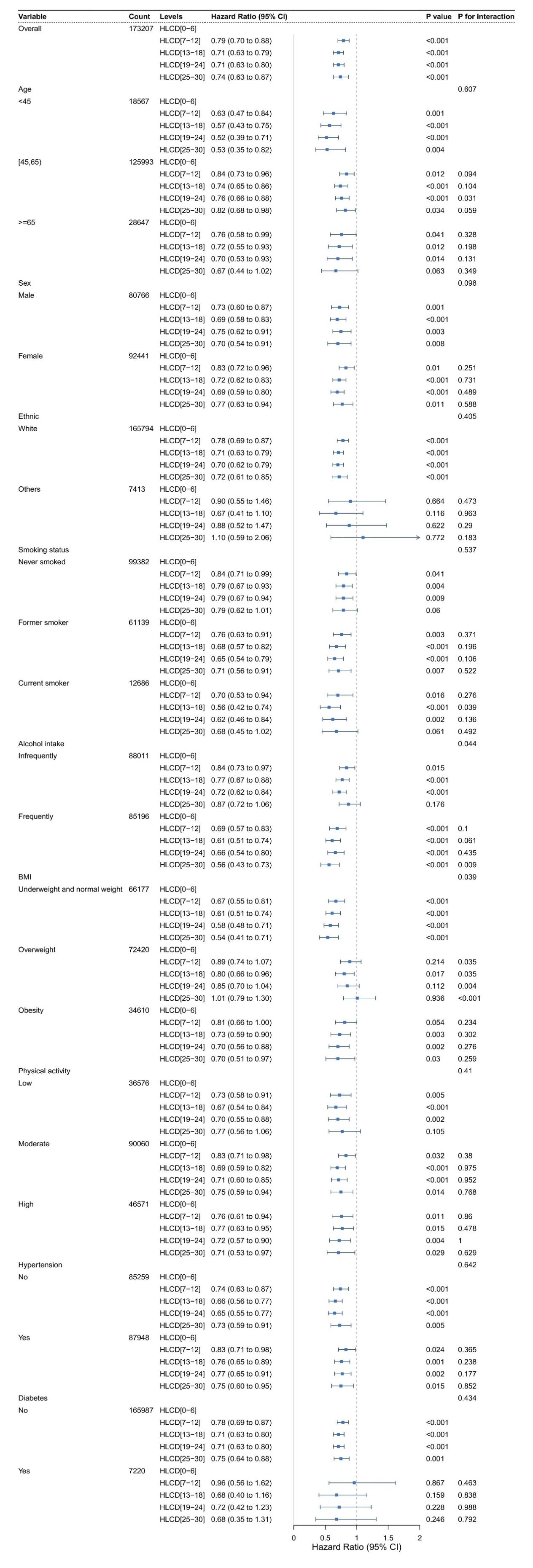
**


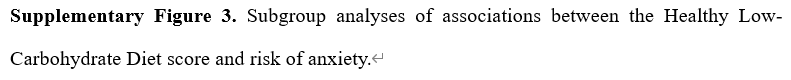
**
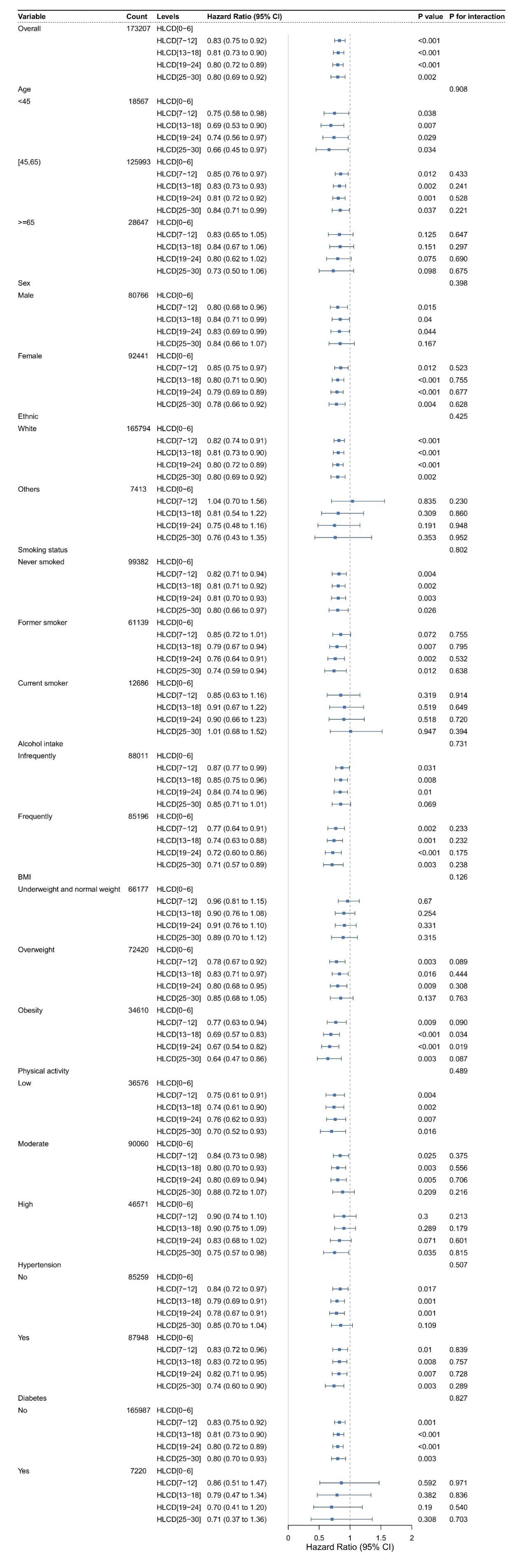
**


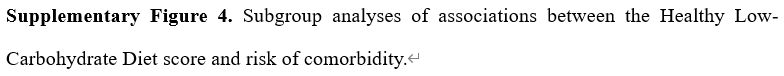
**
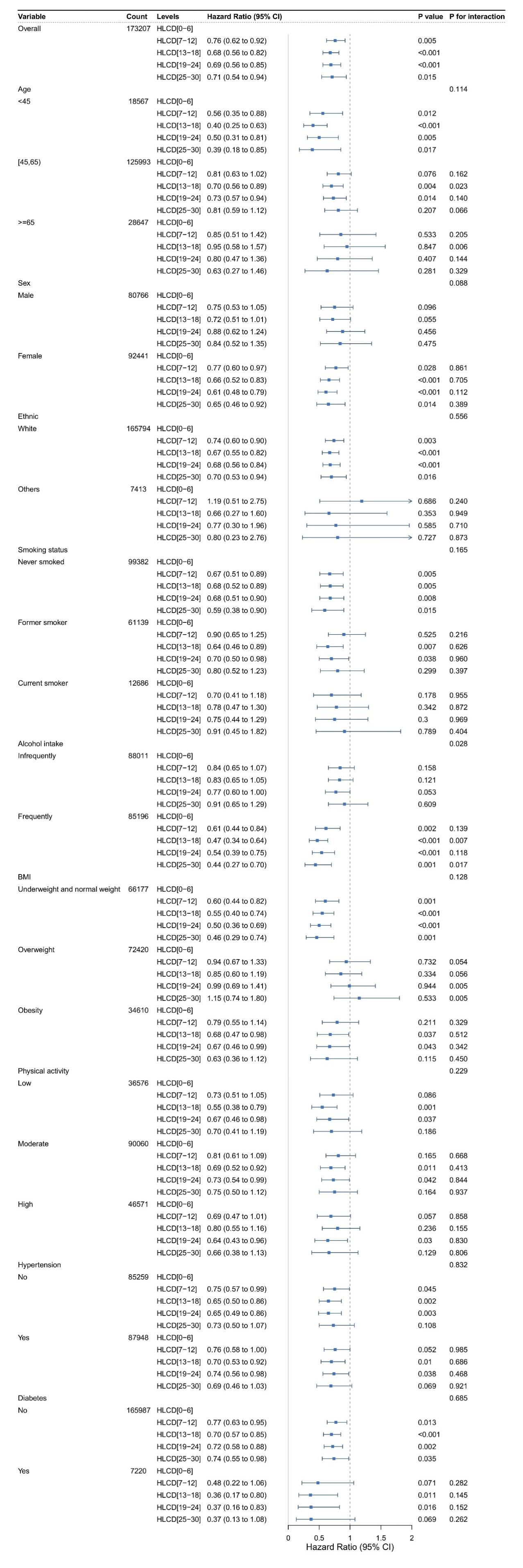
**

**Supplementary Table 1.** Criteria for determining the healthy low-carbohydrate diet (HLCD) score.

| **Points** | **Total Carbohydrate Intake (%)** | **Vegetable Protein Intake (%)** | **Unsaturated Fat Intake (%)** |
| --- | --- | --- | --- |
| 0 | > 60.2 | < 3.8 | < 11.7 |
| 1 | 56.8-60.2 | 3.8-4.3 | 11.7-13.3 |
| 2 | 54.4-56.8 | 4.3-4.6 | 13.3-14.5 |
| 3 | 52.5-54.4 | 4.6-4.9 | 14.5-15.4 |
| 4 | 50.7-52.5 | 4.9-5.2 | 15.4-16.3 |
| 5 | 49.0-50.7 | 5.2-5.5 | 16.3-17.2 |
| 6 | 47.1-49.0 | 5.5-5.8 | 17.2-18.1 |
| 7 | 45.1-47.1 | 5.8-6.2 | 18.1-19.2 |
| 8 | 42.5-45.1 | 6.2-6.7 | 19.2-20.5 |
| 9 | 38.7-42,5 | 6.7-7.5 | 20.5-22.6 |
| 10 | < 38.7 | > 7.5 | > 22.6 |

**Supplementary Table 2.** Associations between the HLCD score and risks of depression and anxiety after excluding participants who only completed the online 24-hour dietary recall questionnaire on 1 occasion (N=106,171).

|  | **N_case_/N_total_** | **Model 1^a^** | | **Model 2^b^** | | **Model 3^c^** | |
| --- | --- | --- | --- | --- | --- | --- | --- |
|  |  | **HR (95% CI)** | **P-value** | **HR (95% CI)** | **P-value** | **HR (95% CI)** | **P-value** |
| Depression | | | | | | | |
| Categories of the HLCD score | | | | | | | |
| ≤ 6 | 146/3,624 | REF |  | REF |  | REF |  |
| 7–12 | 826/28,861 | 0.703(0.590-0.839) | < 0.001 | 0.713(0.598-0.850) | < 0.001 | 0.735(0.616-0.876) | < 0.001 |
| 13–18 | 1,160/44,741 | 0.635(0.535-0.755) | < 0.001 | 0.636(0.535-0.755) | < 0.001 | 0.650(0.547-0.773) | < 0.001 |
| 19–24 | 659/24,384 | 0.662(0.553-0.792) | < 0.001 | 0.645(0.539-0.772) | < 0.001 | 0.649(0.542-0.778) | < 0.001 |
| ≥ 25 | 128/4,561 | 0.688(0.543-0.873) | 0.002 | 0.658(0.519-0.835) | < 0.001 | 0.666(0.525-0.846) | < 0.001 |
| Anxiety | | | | | | | |
| Categories of the HLCD score | | | | | | | |
| ≤ 6 | 170/3,624 | REF |  | REF |  | REF |  |
| 7–12 | 1,113/28,861 | 0.815(0.694-0.958) | 0.013 | 0.827(0.703-0.972) | 0.021 | 0.850(0.723-0.999) | 0.049 |
| 13–18 | 1,733/44,741 | 0.817(0.698-0.957) | 0.012 | 0.818(0.699-0.958) | 0.013 | 0.843(0.719-0.987) | 0.034 |
| 19–24 | 960/24,384 | 0.830(0.705-0.978) | 0.026 | 0.808(0.686-0.951) | 0.010 | 0.824(0.700-0.971) | 0.021 |
| ≥ 25 | 179/4,561 | 0.829(0.672-1.022) | 0.079 | 0.798(0.647-0.985) | 0.035 | 0.812(0.657-1.002) | 0.052 |
| Comorbidity | | | | | | | |
| Categories of the HLCD score | | | | | | | |
| ≤ 6 | 44/3,624 | REF |  | REF |  | REF |  |
| 7–12 | 261/28,861 | 0.739(0.537-1.018) | 0.064 | 0.750(0.545-1.032) | 0.077 | 0.788(0.572-1.085) | 0.144 |
| 13–18 | 379/44,741 | 0.691(0.506-0.945) | 0.020 | 0.692(0.506-0.945) | 0.021 | 0.731(0.535-1.001) | 0.051 |
| 19–24 | 209/24,384 | 0.699(0.505-0.967) | 0.031 | 0.679(0.491-0.940) | 0.020 | 0.707(0.510-0.981) | 0.038 |
| ≥ 25 | 43/4,561 | 0.769(0.505-1.171) | 0.220 | 0.734(0.482-1.117) | 0.149 | 0.765(0.502-1.166) | 0.213 |

Abbreviations: HLCD, Healthy Low-Carbohydrate Diet.

^a^Model 1: unadjusted.

^b^Model 2: adjusted for age, sex, Townsend scores, and ethnicity.

^c^Model 3: additionally adjusted for smoking status, alcohol intake, BMI, physical activity, hypertension, diabetes, and total energy intake on Model .

**Supplementary Table 3.** Associations between the HLCD score and risks of depression and anxiety, redefining follow-up duration using the latest dietary assessment as baseline (N=171,610).

|  | **N_case_/N_total_** | **Model 1^a^** | | **Model 2^b^** | | **Model 3^c^** | |
| --- | --- | --- | --- | --- | --- | --- | --- |
|  |  | **HR (95% CI)** | **P-value** | **HR (95% CI)** | **P-value** | **HR (95% CI)** | **P-value** |
| Depression | | | | | | | |
| Categories of the HLCD score | | | | | | | |
| ≤ 6 | 326/8,860 | REF |  | REF |  | REF |  |
| 7–12 | 1,371/49,473 | 0.770(0.682-0.868) | < 0.001 | 0.782(0.693-0.882) | < 0.001 | 0.807(0.715-0.911) | < 0.001 |
| 13–18 | 1,669/67,214 | 0.698(0.620-0.786) | < 0.001 | 0.702(0.623-0.791) | < 0.001 | 0.723(0.642-0.815) | < 0.001 |
| 19–24 | 983/37,797 | 0.729(0.643-0.826) | < 0.001 | 0.717(0.632-0.813) | < 0.001 | 0.730(0.643-0.828) | < 0.001 |
| ≥ 25 | 220/8,266 | 0.737(0.621-0.874) | < 0.001 | 0.719(0.606-0.854) | < 0.001 | 0.731(0.615-0.868) | < 0.001 |
| Anxiety | | | | | | | |
| Categories of the HLCD score | | | | | | | |
| ≤ 6 | 386/8,860 | REF |  | REF |  | REF |  |
| 7–12 | 1,792/49,473 | 0.852(0.764-0.952) | 0.004 | 0.867(0.777-0.968) | 0.011 | 0.889(0.796-0.992) | 0.036 |
| 13–18 | 2,378/67,214 | 0.843(0.757-0.939) | 0.002 | 0.851(0.764-0.948) | 0.003 | 0.872(0.783-0.972) | 0.013 |
| 19–24 | 1,353/37,797 | 0.851(0.760-0.953) | 0.005 | 0.837(0.747-0.937) | 0.002 | 0.852(0.761-0.955) | 0.006 |
| ≥ 25 | 290/8,266 | 0.822(0.706-0.957) | 0.012 | 0.807(0.693-0.940) | 0.006 | 0.819(0.703-0.954) | 0.010 |
| Comorbidity | | | | | | | |
| Categories of the HLCD score | | | | | | | |
| ≤ 6 | 112/8,860 | REF |  | REF |  | REF |  |
| 7–12 | 456/49,473 | 0.755(0.614-0.929) | 0.008 | 0.770(0.626-0.947) | 0.013 | 0.800(0.650-0.984) | 0.035 |
| 13–18 | 540/67,214 | 0.670(0.547-0.822) | < 0.001 | 0.676(0.551-0.829) | < 0.001 | 0.704(0.573-0.864) | < 0.001 |
| 19–24 | 330/37,797 | 0.726(0.586-0.899) | 0.003 | 0.711(0.574-0.882) | 0.002 | 0.734(0.591-0.910) | 0.005 |
| ≥ 25 | 70/8,266 | 0.691(0.512-0.931) | 0.015 | 0.673(0.499-0.907) | 0.009 | 0.691(0.512-0.932) | 0.016 |

Abbreviations: HLCD, Healthy Low-Carbohydrate Diet.

^a^Model 1: unadjusted.

^b^Model 2: adjusted for age, sex, Townsend scores, and ethnicity.

^c^Model 3: additionally adjusted for smoking status, alcohol intake, BMI, physical activity, hypertension, diabetes, and total energy intake on Model 2.

**Supplementary Table 4.** Associations between the HLCD score and risks of depression and anxiety after excluding depression or anxiety cases that occurred within the first five years of follow-up (N=169,484).

|  | **N_case_/N_total_** | **Model 1^a^** | | **Model 2^b^** | | **Model 3^c^** | |
| --- | --- | --- | --- | --- | --- | --- | --- |
|  |  | **HR (95% CI)** | **P-value** | **HR (95% CI)** | **P-value** | **HR (95% CI)** | **P-value** |
| Depression | | | | | | | |
| Categories of the HLCD score | | | | | | | |
| ≤ 6 | 256/8,718 | REF |  | REF |  | REF |  |
| 7–12 | 1,056/48,833 | 0.726(0.633-0.832) | < 0.001 | 0.737(0.643-0.845) | < 0.001 | 0.759(0.662-0.870) | < 0.001 |
| 13–18 | 1,294/66,428 | 0.650(0.569-0.744) | < 0.001 | 0.654(0.572-0.748) | < 0.001 | 0.671(0.586-0.768) | < 0.001 |
| 19–24 | 775/37,354 | 0.693(0.601-0.798) | < 0.001 | 0.682(0.592-0.785) | < 0.001 | 0.691(0.599-0.796) | < 0.001 |
| ≥ 25 | 158/8,151 | 0.648(0.531-0.790) | < 0.001 | 0.633(0.519-0.771) | < 0.001 | 0.639(0.524-0.780) | < 0.001 |
| Anxiety | | | | | | | |
| Categories of the HLCD score | | | | | | | |
| ≤ 6 | 291/8,718 | REF |  | REF |  | REF |  |
| 7–12 | 1,331/48,833 | 0.805(0.709-0.914) | < 0.001 | 0.815(0.718-0.926) | 0.002 | 0.835(0.735-0.948) | 0.005 |
| 13–18 | 1,835/66,428 | 0.813(0.718-0.920) | 0.001 | 0.818(0.722-0.925) | 0.001 | 0.836(0.738-0.947) | 0.005 |
| 19–24 | 1,033/37,354 | 0.813(0.714-0.926) | 0.002 | 0.798(0.701-0.909) | < 0.001 | 0.810(0.711-0.923) | 0.002 |
| ≥ 25 | 216/8,151 | 0.780(0.654-0.930) | 0.006 | 0.768(0.644-0.916) | 0.003 | 0.776(0.651-0.926) | 0.005 |
| Comorbidity | | | | | | | |
| Categories of the HLCD score | | | | | | | |
| ≤ 6 | 90/8,718 | REF |  | REF |  | REF |  |
| 7–12 | 332/48,833 | 0.649(0.514-0.819) | < 0.001 | 0.660(0.523-0.833) | < 0.001 | 0.687(0.544-0.868) | 0.002 |
| 13–18 | 424/66,428 | 0.606(0.483-0.761) | < 0.001 | 0.610(0.486-0.766) | < 0.001 | 0.636(0.506-0.800) | < 0.001 |
| 19–24 | 255/37,354 | 0.648(0.510-0.824) | < 0.001 | 0.633(0.498-0.805) | < 0.001 | 0.653(0.513-0.832) | < 0.001 |
| ≥ 25 | 53/8,151 | 0.618(0.440-0.868) | 0.005 | 0.601(0.428-0.843) | 0.003 | 0.616(0.438-0.867) | 0.005 |

Abbreviations: HLCD, Healthy Low-Carbohydrate Diet.

^a^Model 1: unadjusted.

^b^Model 2: adjusted for age, sex, Townsend scores, and ethnicity.

^c^Model 3: additionally adjusted for smoking status, alcohol intake, BMI, physical activity, hypertension, diabetes, and total energy intake on Model 2.

**Supplementary Table 5.** Associations between percentages of energy from low-quality carbohydrate and risks of depression and anxiety.

|  | **N_case_/N_total_** | **Model 1^a^** | | **Model 2^b^** | | **Model 3^c^** | |
| --- | --- | --- | --- | --- | --- | --- | --- |
|  |  | **HR (95% CI)** | **P-value** | **HR (95% CI)** | **P-value** | **HR (95% CI)** | **P-value** |
| Depression | | | | | | | |
| Percentages of energy from low-quality carbohydrate | | | | | | | |
| Quintile 1 | 1,065/34,945 | REF |  | REF |  | REF |  |
| Quintile 2 | 998/35,051 | 0.931(0.854-1.015) | 0.103 | 0.940(0.862-1.025) | 0.162 | 0.961(0.881-1.049) | 0.375 |
| Quintile 3 | 1,007/34,914 | 0.942(0.864-1.027) | 0.176 | 0.951(0.872-1.036) | 0.252 | 0.984(0.901-1.074) | 0.711 |
| Quintile 4 | 1,073/34,742 | 1.011(0.929-1.100) | 0.801 | 1.017(0.934-1.107) | 0.692 | 1.045(0.958-1.141) | 0.322 |
| Quintile 5 | 1,141/33,555 | 1.120(1.030-1.218) | 0.008 | 1.117(1.028-1.215) | 0.009 | 1.134(1.039-1.239) | 0.005 |
| Anxiety | | | | | | | |
| Percentages of energy from low-quality carbohydrate | | | | | | | |
| Quintile 1 | 1,395/34,945 | REF |  | REF |  | REF |  |
| Quintile 2 | 1,393/35,051 | 0.994(0.922-1.070) | 0.864 | 0.992(0.921-1.069) | 0.836 | 0.993(0.921-1.070) | 0.846 |
| Quintile 3 | 1,419/34,914 | 1.015(0.943-1.093) | 0.688 | 1.005(0.933-1.082) | 0.894 | 1.002(0.929-1.080) | 0.958 |
| Quintile 4 | 1,459/34,742 | 1.051(0.977-1.131) | 0.184 | 1.036(0.963-1.115) | 0.345 | 1.020(0.946-1.100) | 0.607 |
| Quintile 5 | 1,577/33,555 | 1.186(1.103-1.274) | < 0.001 | 1.157(1.076-1.243) | < 0.001 | 1.127(1.044-1.216) | 0.002 |
| Comorbidity | | | | | | | |
| Percentages of energy from low-quality carbohydrate | | | | | | | |
| Quintile 1 | 329/34,945 | REF |  | REF |  | REF |  |
| Quintile 2 | 321/35,051 | 0.969(0.831-1.130) | 0.687 | 0.976(0.837-1.138) | 0.758 | 0.989(0.848-1.155) | 0.891 |
| Quintile 3 | 330/34,914 | 1.000(0.858-1.165) | 0.996 | 1.002(0.860-1.168) | 0.975 | 1.019(0.873-1.191) | 0.809 |
| Quintile 4 | 355/34,742 | 1.082(0.932-1.258) | 0.301 | 1.081(0.930-1.256) | 0.308 | 1.083(0.928-1.264) | 0.313 |
| Quintile 5 | 378/33,555 | 1.202(1.037-1.393) | 0.015 | 1.187(1.023-1.377) | 0.023 | 1.166(0.997-1.362) | 0.054 |

^a^Model 1: unadjusted.

^b^Model 2: adjusted for age, sex, Townsend scores, and ethnicity.

^c^Model 3: additionally adjusted for smoking status, alcohol intake, BMI, physical activity, hypertension, diabetes, and total energy intake on Model 2.

**Supplementary Table 6.** Associations between percentages of energy from vegetable protein and risks of depression and anxiety.

|  | **N_case_/N_total_** | **Model 1^a^** | | **Model 2^b^** | | **Model 3^c^** | |
| --- | --- | --- | --- | --- | --- | --- | --- |
|  |  | **HR (95% CI)** | **P-value** | **HR (95% CI)** | **P-value** | **HR (95% CI)** | **P-value** |
| Depression | | | | | | | |
| Percentages of energy from vegetable protein | | | | | | | |
| Quintile 1 | 1,225/33,794 | REF |  | REF |  | REF |  |
| Quintile 2 | 1,038/34,738 | 0.818(0.753-0.889) | < 0.001 | 0.827(0.762-0.899) | < 0.001 | 0.854(0.786-0.928) | < 0.001 |
| Quintile 3 | 961/35,150 | 0.747(0.686-0.813) | < 0.001 | 0.754(0.693-0.821) | < 0.001 | 0.786(0.722-0.856) | < 0.001 |
| Quintile 4 | 1,005/35,058 | 0.783(0.720-0.851) | < 0.001 | 0.787(0.724-0.856) | < 0.001 | 0.821(0.755-0.894) | < 0.001 |
| Quintile 5 | 1,055/34,467 | 0.839(0.773-0.911) | < 0.001 | 0.824(0.758-0.894) | < 0.001 | 0.854(0.785-0.929) | < 0.001 |
| Anxiety | | | | | | | |
| Percentages of energy from vegetable protein | | | | | | | |
| Quintile 1 | 1,560/33,794 | REF |  | REF |  | REF |  |
| Quintile 2 | 1,402/34,738 | 0.868(0.808-0.933) | < 0.001 | 0.867(0.807-0.932) | < 0.001 | 0.882(0.820-0.948) | < 0.001 |
| Quintile 3 | 1,361/35,150 | 0.832(0.773-0.894) | < 0.001 | 0.828(0.770-0.890) | < 0.001 | 0.845(0.786-0.909) | < 0.001 |
| Quintile 4 | 1,419/35,058 | 0.870(0.809-0.934) | < 0.001 | 0.861(0.801-0.925) | < 0.001 | 0.877(0.816-0.943) | < 0.001 |
| Quintile 5 | 1,501/34,467 | 0.939(0.875-1.008) | 0.084 | 0.909(0.847-0.976) | 0.009 | 0.918(0.854-0.987) | 0.021 |
| Comorbidity | | | | | | | |
| Percentages of energy from vegetable protein | | | | | | | |
| Quintile 1 | 393/33,794 | REF |  | REF |  | REF |  |
| Quintile 2 | 333/34,738 | 0.819(0.708-0.948) | 0.007 | 0.825(0.713-0.955) | 0.010 | 0.854(0.738-0.989) | 0.035 |
| Quintile 3 | 291/35,150 | 0.706(0.607-0.822) | < 0.001 | 0.710(0.610-0.827) | < 0.001 | 0.740(0.636-0.862) | < 0.001 |
| Quintile 4 | 365/35,058 | 0.888(0.770-1.024) | 0.103 | 0.888(0.770-1.025) | 0.104 | 0.923(0.799-1.066) | 0.277 |
| Quintile 5 | 331/34,467 | 0.822(0.710-0.951) | 0.008 | 0.799(0.690-0.925) | 0.003 | 0.819(0.705-0.951) | 0.009 |

^a^Model 1: unadjusted.

^b^Model 2: adjusted for age, sex, Townsend scores, and ethnicity.

^c^Model 3: additionally adjusted for smoking status, alcohol intake, BMI, physical activity, hypertension, diabetes, and total energy intake on Model 2.

**Supplementary Table 7.** Associations between percentages of energy from unsaturated fat and risks of depression and anxiety.

|  | **N_case_/N_total_** | **Model 1^a^** | | **Model 2^b^** | | **Model 3^c^** | |
| --- | --- | --- | --- | --- | --- | --- | --- |
|  |  | **HR (95% CI)** | **P-value** | **HR (95% CI)** | **P-value** | **HR (95% CI)** | **P-value** |
| Depression | | | | | | | |
| Percentages of energy from unsaturated fat | | | | | | | |
| Quintile 1 | 1,097/34,512 | REF |  | REF |  | REF |  |
| Quintile 2 | 1,025/34,890 | 0.919(0.844-1.001) | 0.053 | 0.918(0.843-0.999) | 0.049 | 0.923(0.847-1.006) | 0.068 |
| Quintile 3 | 1,009/34,906 | 0.903(0.829-0.984) | 0.019 | 0.894(0.820-0.973) | 0.010 | 0.891(0.818-0.972) | 0.009 |
| Quintile 4 | 1,081/34,703 | 0.974(0.895-1.059) | 0.537 | 0.953(0.876-1.036) | 0.260 | 0.934(0.858-1.018) | 0.12 |
| Quintile 5 | 1,072/34,196 | 0.981(0.902-1.067) | 0.655 | 0.935(0.859-1.017) | 0.118 | 0.895(0.821-0.976) | 0.012 |
| Anxiety | | | | | | | |
| Percentages of energy from unsaturated fat | | | | | | | |
| Quintile 1 | 1,485/34,512 | REF |  | REF |  | REF |  |
| Quintile 2 | 1,370/34,890 | 0.907(0.843-0.976) | 0.009 | 0.903(0.839-0.972) | 0.007 | 0.898(0.834-0.967) | 0.004 |
| Quintile 3 | 1,420/34,906 | 0.939(0.873-1.009) | 0.088 | 0.924(0.859-0.994) | 0.034 | 0.911(0.846-0.981) | 0.013 |
| Quintile 4 | 1,458/34,703 | 0.970(0.902-1.043) | 0.409 | 0.944(0.878-1.015) | 0.117 | 0.918(0.852-0.988) | 0.022 |
| Quintile 5 | 1,510/34,196 | 1.021(0.951-1.097) | 0.566 | 0.966(0.899-1.038) | 0.352 | 0.927(0.861-0.997) | 0.042 |
| Comorbidity | | | | | | | |
| Percentages of energy from unsaturated fat | | | | | | | |
| Quintile 1 | 361/34,512 | REF |  | REF |  | REF |  |
| Quintile 2 | 308/34,890 | 0.839(0.720-0.976) | 0.023 | 0.836(0.718-0.974) | 0.021 | 0.841(0.722-0.980) | 0.027 |
| Quintile 3 | 340/34,906 | 0.924(0.796-1.071) | 0.293 | 0.911(0.785-1.056) | 0.217 | 0.908(0.782-1.055) | 0.209 |
| Quintile 4 | 361/34,703 | 0.986(0.852-1.141) | 0.852 | 0.959(0.829-1.110) | 0.576 | 0.939(0.809-1.089) | 0.405 |
| Quintile 5 | 343/34,196 | 0.952(0.821-1.103) | 0.511 | 0.897(0.773-1.040) | 0.150 | 0.857(0.736-0.997) | 0.046 |

^a^Model 1: unadjusted.

^b^Model 2: adjusted for age, sex, Townsend scores, and ethnicity.

^c^Model 3: additionally adjusted for smoking status, alcohol intake, BMI, physical activity, hypertension, diabetes, and total energy intake on Model 2.

**Supplementary Table 8.** Associations between the HLCD score and inflammatory indicators.

|  | **Lymphocyte count** | | **Neutrophil count** | | **Platelet count** | | **White blood cell count** | | **CRP** | | **NLR** | | **INFLA** | |
| --- | --- | --- | --- | --- | --- | --- | --- | --- | --- | --- | --- | --- | --- | --- |
|  | **β (95% CI)** | **P-value** | **β (95% CI)** | **P-value** | **β (95% CI)** | **P-value** | **β (95% CI)** | **P-value** | **β (95% CI)** | **P-value** | **β (95% CI)** | **P-value** | **β (95% CI)** | **P-value** |
| Categories of the HLCD score | | | | | | | | | | | | | | |
| ≤ 6 | REF |  | REF |  | REF |  | REF |  | REF |  | REF |  | REF |  |
| 7–12 | -0.034(-0.058, -0.011) | < 0.001 | -0.011(-0.034, 0.013) | 0.377 | -0.017(-0.040, 0.007) | 0.164 | -0.028(-0.052, -0.005) | 0.018 | -0.045(-0.067, -0.023) | < 0.001 | 0.007(-0.002, 0.016) | 0.152 | -0.195(-0.330, -0.059) | 0.005 |
| 13–18 | -0.053(-0.076, -0.030) | 0.004 | -0.016(-0.039, 0.007) | 0.175 | -0.016(-0.039, 0.007) | 0.184 | -0.045(-0.068, -0.022) | < 0.001 | -0.060(-0.082, -0.039) | < 0.001 | 0.010(0.001, 0.019) | 0.023 | -0.242(-0.375, -0.108) | < 0.001 |
| 19–24 | -0.051(-0.075, -0.027) | < 0.001 | -0.022(-0.046, 0.002) | 0.076 | -0.023(-0.047, 0.002) | 0.066 | -0.052(-0.076, -0.028) | < 0.001 | -0.080(-0.102, -0.057) | < 0.001 | 0.008(-0.001, 0.017) | 0.097 | -0.349(-0.488, -0.210) | < 0.001 |
| ≥ 25 | -0.090(-0.121, -0.058) | < 0.001 | -0.044(-0.075, -0.012) | 0.006 | -0.034(-0.065, -0.003) | 0.032 | -0.092(-0.123, -0.061) | < 0.001 | -0.124(-0.153, -0.096) | < 0.001 | 0.012(0.000, 0.025) | 0.043 | -0.558(-0.737, -0.378) | < 0.001 |

Using multiple linear regression models adjusted for age, sex, Townsend scores, ethnicity, smoking status, alcohol intake, BMI, physical activity, hypertension, diabetes, and total energy intake. Abbreviations: HLCD, Healthy Low-Carbohydrate Diet; CRP, C-reactive protein; NLR, Neutrophil-to-Lymphocyte Ratio; INFLA, Low-grade chronic inflammation score.

**Supplementary Table 9.** Associations between inflammatory indicators and risks of depression and anxiety.

|  | **Depression** | | **Anxiety** | | **Comorbidity** | |
| --- | --- | --- | --- | --- | --- | --- |
|  | **HR (95% CI)** | **P-value** | **HR (95% CI)** | **P-value** | **HR (95% CI)** | **P-value** |
| Lymphocyte count | 1.023(0.992-1.054) | 0.143 | 1.030(1.004-1.057) | 0.023 | 1.036(0.983-1.093) | 0.189 |
| Neutrophil count | 1.086(1.053-1.120) | < 0.001 | 1.090(1.062-1.119) | < 0.001 | 1.115(1.055-1.177) | < 0.001 |
| Platelet count | 1.020(0.989-1.051) | 0.205 | 1.018(0.992-1.045) | 0.182 | 0.991(0.940-1.045) | 0.747 |
| White blood cell count | 1.088(1.056-1.122) | < 0.001 | 1.095(1.067-1.124) | < 0.001 | 1.115(1.058-1.177) | < 0.001 |
| CRP | 1.077(1.042-1.112) | < 0.001 | 1.045(1.017-1.074) | 0.002 | 1.105(1.044-1.170) | < 0.001 |
| NLR | 1.140(1.054-1.234) | 0.001 | 1.133(1.059-1.211) | < 0.001 | 1.173(1.022-1.347) | 0.024 |
| INFLA | 1.016(1.011-1.022) | < 0.001 | 1.015(1.011-1.020) | < 0.001 | 1.020(1.010-1.029) | < 0.001 |

Using Cox models adjusted for age, sex, Townsend scores, ethnicity, smoking status, alcohol intake, BMI, physical activity, hypertension, diabetes, and total energy intake. Abbreviations: CRP, C-reactive protein; NLR, Neutrophil-to-Lymphocyte Ratio; INFLA, Low-grade chronic inflammation score.

**References**

1. Townsend P, Phillimore P, Beattie A. Health and Deprivation: Inequality and the North. London: Routledge; 2023. https://doi.org/10.4324/9781003368885.

2. Jarman B, Townsend P, Carstairs V. Deprivation indices. BMJ. 1991;303:523. https://doi.org/10.1136/bmj.303.6801.523-a.

3. Ainsworth BE, Haskell WL, Whitt MC, Irwin ML, Swartz AM, Strath SJ, et al. Compendium of physical activities: an update of activity codes and MET intensities. Med Sci Sports Exerc. 2000;32 9 Suppl:S498-504. https://doi.org/10.1097/00005768-200009001-00009.
